# Impact and Novel Perspective of Immune Checkpoint Inhibitors in Patients with Early and Intermediate Stage HCC

**DOI:** 10.3390/cancers14143332

**Published:** 2022-07-08

**Authors:** Luca Marzi, Andrea Mega, Stefano Gitto, Filippo Pelizzaro, Andreas Seeber, Gilbert Spizzo

**Affiliations:** 1 Department of Gastroenterology, Bolzano Regional Hospital (SABES-ASDAA), 39100 Bolzano-Bozen, Italy; luca.marzi@sabes.it (L.M.); andrea.mega@sabes.it (A.M.); 2 Department of Experimental and Clinical Medicine, University of Firenze, 50134 Firenze, Italy; stefano.gitto@unifi.it; 3 Department of Gastroenterology, Medical University of Padova, 35128 Padova, Italy; filippo.pelizzaro@aopd.veneto.it; 4 Department of Hematology and Oncology, Comprehensive Cancer Center Innsbruck, 6020 Innsbruck, Austria; 5 Department of Internal Medicine, Oncologic Day Hospital, Hospital of Bressanone (SABES-ASDAA), 39042 Bressanone-Brixen, Italy

**Keywords:** targeted therapy, locoregional treatment, immunotherapy, multimodality therapy, immune checkpoint inhibitors, hepatocellular carcinoma

## Abstract

**Simple Summary:**

The prognostic evaluation and therapeutic management of hepatocarcinoma (HCC) is based on the Barcelona Liver Cancer Clinic (BCLC). In the past years, immunotherapy has become a mainstay of first-line treatment in advanced HCC and further treatment options are underway. Beyond this, the scientific community is more and more focusing on immunotherapeutic approaches in earlier HCC stages. The purpose of this review is to describe the rationale for immunotherapeutic approaches and the studies conducted with immunotherapy in patients with early and intermediate stage HCC.

**Abstract:**

Surgery and radiofrequency ablation remain the gold standard to achieve cure in patients with hepatocellular carcinoma (HCC). After a decade in which only sorafenib was available for advanced and metastatic HCC, the emergence of other molecularly targeted drugs and immune checkpoint inhibitors (ICIs) has significantly improved the patients` prognosis. In particular, the use of ICIs has shown promising results and has revolutionized the treatment algorithm in HCC patients. Indeed, preclinical and clinical data have documented a high density of immunosuppressive cells and an increased expression of the programmed death-1 (PD-1) receptor and cytotoxic T-cell associated protein-4 (CTLA-4) in HCC. However, despite these observations, no validated biomarker is available and the molecular groundwork responsible for response to ICIs remains elusive. The anti-CTLA4 monoclonal antibody tremelimumab and the anti-PD-1 monoclonal antibodies nivolumab and pembrolizumab were the first ICIs to be tested in HCC. Recently, the combination of the anti-programmed death-ligand 1 (PD-L1) inhibitor atezolizumab and the anti-vascular endothelial growth factor (VEGF) antibody bevacizumab demonstrated an improvement in patient outcome compared to sorafenib, becoming the standard of care in the frontline setting of advanced disease. Other immunotherapeutic agents such as pembrolizumab or the combination nivolumab-ipilimumab have shown promising results that have to be confirmed in phase III studies. Currently, the combination of different ICIs (i.e., ipilimumab, durvalumab) and anti-angiogenic agents (i.e., regorafenib, lenvatinib) is currently being tested in several trials and will hopefully revolutionize the treatment of HCC. To date, numerous studies are underway evaluating ICIs in adjuvant and neoadjuvant settings to improve survival in early and intermediate stages. Thus, this review focuses on the rationale for ICIs and their potential use for early or intermediate HCC stages.

## 1. Introduction

Hepatocellular carcinoma (HCC) is the third cause of cancer-related death worldwide in 2020 and the sixth most frequent cancer [1]. The prognosis and treatment of HCC are based on the Barcelona Liver Cancer Clinic (BCLC), where the disease is divided into four stages upon which treatment decisions are based [2]. The BCLC staging system includes prognostic variables related to tumor burden (size and numbers of nodules, portal or extrahepatic invasion), liver function (according to Child-Pugh classification, albumin, bilirubin, prothrombin time, hepatic encephalopathy, and ascites) and general health status (according to the Eastern Cooperative Oncology Group [ECOG] classification) [2]. The stage is therefore directly related to the proposed treatment strategy [2]. 

In general, patients with very early or early-stage HCC (BCLC 0 single tumor nodule < 2 cm or BCLC A single nodule or 2–3 nodules < 3 cm, preserved liver function and PS 0, respectively) are preferred candidates for curative treatments such as liver resection (LR), orthotopic liver transplant (LT) or local ablation (LA). Intermediate-stage patients (BCLC B multifocal or unresectable tumors, preserved liver function, and PS 0) are candidates for transarterial chemoembolization (TACE) and advanced hepatocellular carcinoma (BCLC C tumor with portal invasion or extrahepatic infiltration, preserved liver function, PS 1–2) is treated with systemic therapy [2]. End-stage patients (BCLC D) who have end-stage liver function and PS 3–4 are candidates for best supportive care (BSC) [2].

Despite the development of surveillance programs, only about 30% of patients are in the early stage (BCLC A) and can undergo curative therapies [3]. Although liver resection is the cornerstone of curative treatment for HCC, the risk of recurrence is high reaching 70% of cases at 5 years [2,4].

As mentioned above, in the intermediate stage (BCLC B), the recommended treatment is TACE [5], which can lead to survival rates of 82%, 47%, and 26% at 1, 3, and 5 years, respectively [5]. Additionally, TACE used in HCC patients with a tumor size of less than or equal to 5 cm (early stage) resulted in 1, 3, and 5-year overall survival (OS) rates of 91%, 66%, and 52, respectively [6].

Systemic therapy is the main treatment for patients with HCC in BCLC C [7], although, in clinical practice, a subset of patients within BCLC-B with diffuse, infiltrative, and extensive liver cancer involvement not benefiting from TACE should be treated with systemic treatment [8]. For about 10 years, the only available systemic treatment for stage C has been sorafenib [9]. In 2019, the Tyrosine Kinase Inhibitor (TKI) lenvatinib was approved as an alternative to sorafenib in the first-line treatment for HCC [10]. Recently, other TKIs or monoclonal antibodies that target angiogenesis were approved after failing on sorafenib, namely, regorafenib, cabozantinib, and ramucirumab [11,12,13]. More recently, immunotherapy has entered the field of HCC treatment [14]. A new therapeutic combination in the advanced phase (Atezolizumab-Bevacizumab—Imbrave 150 Trial) was recently approved [15]. This combination showed superior survival compared to sorafenib, while there are no data compared to lenvatinib. Another more recent combination (tremelimumab and durvalumab) showed superiority compared to sorafenib, adding another first-line treatment option [16].

Since immunotherapy has emerged to be the standard first-line treatment for patients with advanced HCC, the use of immunotherapy (neoadjuvant or adjuvant) has prompted clinical trials in the initial stages of HCC and (in association with local therapies) in the intermediate stages. The introduction of immunotherapy even in the early or intermediate stages remains the subject of continuous research. 

To date, HCC treatment guidelines do not recommend the use of immunotherapy in the very early, early, and intermediate stages. Furthermore, although there is a rationale, there are still only little published data [17].

Considering the very promising role of ICI as a combination therapy with local treatments in early and intermediate stages, the aim of this review is to summarize the existing data concerning the combination of potentially curative treatments and TACE with immunotherapy as adjuvant or neoadjuvant treatment in early or intermediate stage HCC.

### 1.1. The Immune System and Immune Checkpoint Inhibitors (ICIs)

The immune system plays a dual role in the development and progression of liver cancer: it can destroy or inhibit cancer cells by suppressing tumor growth, but it can also select tumor cell clones and promote their progression [18]. 

Tumor progression is mediated by liver antigen tolerance (the liver is exposed to many exogenous antigens), immunosuppression of chronic inflammation, and HCC-dependent immune tolerance [19]. Liver tolerance appears to be determined by the release of cytokines such as interleukin 10 (IL-10) and tumor growth factor-beta (TGF-b), by Kupffer cells (KC), and by liver sinusoidal endothelial cells (LSEC) [19]. Moreover, overexpression of immune checkpoints (cell death protein 1 (PD-1), and its ligand) on hepatocytes, hepatic stellate cells, KC, LSEC, and intrahepatic leukocytes [20] and the activation of cytotoxic T-cell-associated protein 4 (CTLA-4) on the surface of T regulatory cells (Treg) [21] appears to further contribute to tumor progression.

The reason for targeting these proteins is that CTLA-4 is a negative co-stimulation regulator that activates T cells after recognition of its tumor antigen presented by an antigen-presenting cell (APC). T-cell receptor antigen recognition (TCR) results in the expression of the PD-1 receptor and the production of interferon-gamma (IFN-γ) which causes reactive expression of PD-L1, that finally deactivates the antitumor responses of the T cells [22].

Hence, blocking the PD-1/PD-L1 axis causes T cell proliferation and infiltration into the tumor, inducing a cytotoxic T cell response leading to objective tumor response [22]. The synergy between the blockade of CTLA-4 and PD-1 appears to be determined by further suppression of inhibitory Treg cells [23].

Following the increase in knowledge of the immune system, strategies have been developed to activate the immune response against tumors. The first approach to cancer treatment was the administration of interleukin-2 (IL-2), capable of stimulating the proliferation of T-lymphocytes. However, the first generation of drugs was burdened with low response rates and numerous side effects [24]. Inhibitory checkpoints commonly expressed on activated T lymphocytes are the most effective approach for activating antitumor immune responses [24].

The efficacy and safety data of monotherapy of ICIs have not shown an increase in overall survival (OS), although results from various early-stage trials are very promising [23]. However, other outcome variables such as tolerability were better than standard Tyrosine Kinase Inhibitor (TKI) therapies, highlighting the potential benefit of ICIs in the treatment of HCC. 

### 1.2. The Rationale for the Association of Immunotherapy and Surgical or Locoregional Treatments 

The rationale for combining immune checkpoint inhibitors (ICIs) with surgical or locoregional treatments among patients with HCC is based on the ability of these therapies to release tumor-associated antigens from apoptotic or necrotic HCC tissue [25]. A randomized controlled trial comparing sorafenib and placebo as adjuvant therapy after liver resection or ablation (STORM study) did not demonstrate any positive effects [26]. 

Locoregional therapy can promote the immune response in patients with HCC. Studies in animal models have documented the activation of tumor-specific T cells and dendritic cells (DC) after radiofrequency [27]. Microwave ablation (MWA) can cause an increase in CD3+, CD4+, and IL-12 cells and a decrease in IL-4 and IL-10 [28]. A study has shown that the overall function of anti-alpha-fetoprotein (AFP) CD4(+) T cells may improve after TACE/TAE. This study suggests that general T-cell immunosuppression can be reversed after locoregional treatments and provides a rationale for combining embolization with immunotherapy in patients with liver tumors [29]. Carcinoembryonic antigen (Glypican-3 (GPC3)) is a target for the anti-HCC response. Another study attempted to compare the induction of the GPC3 T-cell-mediated immune response after radiofrequency ablation (RFA), surgical resection, and TACE in patients with HCC and tumor-bearing mice. After RFA and TACE, compared to resection, an increase in circulating GPC3-specific cytotoxic T lymphocytes (CTLs) was observed [30]. Another locoregional approach is radioembolization using intra-arterial Y90. A study by Chew V et al. showed signs of local immune activation after TARE such as increased expression of granzyme B (GB) and infiltration of CD8+ T cells, CD56+ NK cells, and CD8+ CD56+ NKT cells [31]. 

However, some studies have shown that the T lymphocyte response is not sufficient to prevent relapse after MWA [32]. In a preclinical study conducted in the HCC animal model, Hepa1–6 mice were treated with MWA and immunotherapy. This study showed that MWA combined with anti-PD-1/anti-CTLA-4 increased the survival time and protected the mice from tumor recurrence, through the increase of Th1-type cytokines and intratumoral infiltration of Th1 cell response [33]. According to the authors, IFN-γ, IL-18, IL2, and Th1-type cytokines were upregulated in the combination-treated group and IL-4 and IL-10, Th2-type cytokines were downregulated. Furthermore, the cytokines IL-18 and IL-2 have proliferating and activating effects on peripheral blood mononuclear cells, differentiation effects of T lymphocytes into Th1 cells, and stimulation of IFN-γ production by Th1 cells [33]. One approach using autologous cytokine-induced killer cells for HCC in 230 patients after resection or ablation showed promising results [34], but the lack of subsequent validation trials has probably hampered its clinical approval. Another study that included 150 patients that were resected for HCC and were treated with autologous lymphocytes showed an improvement in recurrence-free survival but not overall survival [35]. In a study of 19 evaluable patients treated with tremelimumab and undergoing subtotal RFA or chemoablation (day 36), five patients (26.3%) achieved a partial response. The six- and 12-month progression-free survival rates were 57.1% and 33.1%, respectively, with a median time to tumor progression of 7.4 months [36]. An interesting hypothesis to explain the efficacy of ICI in patients treated with locoregional therapies is that the release of tumor antigens following the necrosis of neoplastic cells caused by ablation or chemoembolization can activate the immune system, therefore, increasing the activity of the ICIs. This, in turn, boosts the activation of tumor-specific APCs and CTLs, resulting in an immunological synergy [37]. A study in patients treated with RFA or TACE showed that tumor-associated antigens (glypican-3, NY-ESO-1, and MAGE-1)-specific CD8^+^ T cell responses suppress the recurrence of HCC, and that c-TACE-induced tumor-specific response [38].

However, the use of immunotherapeutic approaches in these low disease burden situations might be the ideal setting compared to high-burden diseases [39]. Recent data reported a manageable adverse events profile of immunotherapy in HCC patients [40]. This retrospective cohort study of patients with different neoplasms undergoing thermal ablation, embolization, or selective internal radiotherapy (SIRT) and anti-PD-1/PD-L1 agents (monotherapy with pembrolizumab, nivolumab and atezolizumab, and ipilimumab plus nivolumab) ≤ 90 days before or ≤30 days after the interventional procedure, showed no unmanageable or unexpected toxicity [40]. Moreover, there are some attempts for combining immunotherapy with surgical and locoregional therapies in a neoadjuvant approach [41]. The neoadjuvant approach offers the chance to test the sensibility of the tumor in vivo and may downstage the tumor to permit a better resectability [42] but results of phase III trials are lacking.

## 2. Methods

ClinicalTrials.gov was last queried on 31 December 2021, for the terms “hepatocellular carcinoma”, “early stage”, “intermediate stage” and “immunotherapy”. Studies in patients undergoing liver resection, radiofrequency ablation (RFA), microwave ablation (MWA), and TACE were included. Studies enrolling patients with BCLC stage C HCC, extrahepatic or lymph node metastases, vascular invasion or portal vein thrombosis, prior systemic therapy including sorafenib, or chemotherapy were excluded. We analyzed one hundred eleven studies conducted in patients undergoing liver resection, RFA, MWA, stereotaxic body radiotherapy, radioembolization, TACE, TAE, and DEB-TACE. At the same time, we excluded the studies enrolling patients with BCLC stage C HCC, extrahepatic or lymph node metastases, vascular invasion or portal vein thrombosis, prior systemic therapy including sorafenib, or chemotherapy. Finally, studies without or unknown results were excluded. In the end, the present review article revised a total of 34 studies.

### 2.1. Ongoing Trials Evaluating Immune Checkpoint Inhibition before Curative Treatment Modalities 

A multicenter phase Ib safety and expansion trial (PRIME-HCC–NCT03682276) was designed to evaluate the safety and bioactivity of the nivolumab/ipilimumab combination before liver resection (LR) in HCC [41]. The objectives of the study were to evaluate the safety, tolerability of the nivolumab/ipilimumab combination before LR, as well as objective response (ORR) and pathological response rates. The results of this study will help to define the role of the neoadjuvant combination nivolumab/ipilimumab in patients undergoing LR [41]. 

Many phase 1 or 2 studies investigate the role of ICIs in the neoadjuvant setting: the single-arm phase 1b study of cabozantinib plus nivolumab (CaboNivo) in patients with locally advanced HCC followed by LR (NCT03299946), the study nivolumab with or without relatlimab for patients with potential LR (NCT04658147), the study of nivolumab, fluorouracil, and interferon-alpha 2b in patients with fibrolamellar cancer that are unresectable (NCT04380545), the effect of nivolumab and pegargiminase (ADI-PEG 20) before LR (NCT04965714), the study of treatment with nivolumab in patients with advanced HCC treated by electroporation (NCT03630640), the study of atezolizumab and bevacizumab before LR (NCT04721132), the study of pembrolizumab in preventing the recurrence of HCC when administered before LR or ablation (NCT03337841), and others (Table 1) [43]. 

Table 1 describes ongoing clinical trials of ICIs before curative treatments (LR or Ablation) for stage 0/A HCC BCLC. 

### 2.2. Ongoing Trials Evaluating Immune Checkpoint Inhibition after Curative Treatment Modalities 

Various studies are ongoing evaluating the effect of adjuvant immunotherapy after curative treatment modalities. One example is the phase 2 study that investigates the role of ICIs in the adjuvant setting of pembrolizumab in preventing the recurrence of HCC when administered after LR or ablation (NCT03337841).

Moreover, phase 3 studies in the adjuvant setting are ongoing, including the placebo-controlled CheckMate 9DX trial (NCT03383458) investigating the recurrence-free survival (RFS) of nivolumab compared to placebo in patients who have undergone LR or have achieved a complete response (CR) after ablation [44] and IMbrave050 (NCT04102098) trial evaluating the efficacy and safety of adjuvant therapy with atezolizumab plus bevacizumab in patients with completely resected or ablated HCC [45].

The EMERALD-2 study (NCT03847428) evaluates the efficacy and safety of durvalumab-bevacizumab or durvalumab as monotherapy or placebo conducted in patients with HCC at high risk of relapse after curative liver resection or ablation [46]. The KEYNOTE-937 (NCT03867084) evaluates the safety and efficacy of pembrolizumab versus placebo in patients with a complete radiological response after liver resection or local ablation and the clinical study JUPITER-04 (NCT03859128) evaluates the role of toripalimab in improving relapse-free survival (RFS) versus placebo in patients undergoing complete LR [43,44,45,46,47].

Table 2 describes ongoing clinical trials of ICIs after treatment options for stage 0/A HCC BCLC (LR or Ablation).

### 2.3. Ongoing Trials Evaluating Immune Checkpoint Inhibitors in Intermediate Stage

As noted above, although systemic therapy is recommended for intermediate-stage patients not susceptible to TACE, there are not many studies available up to now. A preliminary study examined the therapeutic efficacy of atezolizumab plus bevacizumab treatment in this situation. After enrolling 95 Japanese patients it was concluded that immunotherapy treatment showed a favorable therapeutic response with an objective response rate (ORR)/control rate disease (DCR) at six weeks of RECIST and mRECIST of 17.7%/84.7% and 42.5%/86.2%, respectively [17]. 

Two ongoing Phase 3 studies (NCT04712643 and ABC-HCC, NCT04803994) aim to test the efficacy and safety of atezolizumab in combination with bevacizumab compared to TACE in patients with intermediate-stage liver cancer. RENOTACE (NCT04777851) is a Phase III study designed to evaluate the efficacy and safety of regorafenib and nivolumab (Rego-Nivo) versus TACE in the intermediate-stage HCC. Table 3 describes current and ongoing studies of immunotherapy for intermediate stage (BCLC B).

### 2.4. Ongoing Trials Evaluating Immune Checkpoint Inhibitors in Combination with Transarterial Treatments

The ongoing Phase 1b PETAL study (NCT03397654) of pembrolizumab after TACE has the primary objective of determining safety and tolerability in patients with intermediate-stage HCC, while the secondary objective is to evaluate progression-free survival rates in terms of Modified Response Evaluation in the mRECIST Criteria. The IMMUTACE (NCT03572582) study evaluates the safety and efficacy of the anti-PD-1 antibody nivolumab in combination with TACE. The study has an actual enrollment of 49 participants. 

Phase 2–3 studies are ongoing, including NCT04174781, NCT04268888 (TACE-3), and NCT04340193 evaluating the OS and Time to TACE Progression (TTTP) of nivolumab or nivolumab/ipilimumab in combination with TACE/TAE, NCT03638141 evaluating the ORR for hepatectomy of durvalumab and tremelimumb in combination with TACE, NCT04340193 (CheckMate 74W) evaluating the safety and tolerability of nivolumab with and without ipilimumab in combination with TACE.

Ischemic cell damage due to TACE can increase the levels of vascular endothelial growth factors (VEGF). For this reason, several therapeutic combinations are being tested: DEB-TACE plus lenvatinib or sorafenib or PD-1 inhibitor (NCT04229355), TACE plus durvalumab and bevacizumab (EMER-ALD-1 study, NCT03778957), and lenvatinib and pembrolizumab in combination with TACE, versus TACE plus placebo (NCT04246177).

Preclinical studies have suggested a synergy of antitumor activity between radiotherapy and ICIs, which is currently under clinical investigation (NCT01935921). A study conducted by Tai D et al. (NCT03033446), aimed to investigate the safety and efficacy of SIRT followed by nivolumab. In this study, 40 patients with an advanced stage, not suitable for curative surgery were enrolled, of which 36 patients (90 %) received Y90 radioembolization followed by nivolumab [48]. One patient (3%) showed a complete response, and ten patients (28%) had a partial response [48]. 

The other two Phase 2 studies tested the safety and anticancer efficacy of the combination of durvalumab and tremelimumab with TACE, Y-90 SIRT, and SBRT.

In the IMMUWIN study (NCT04522544), patients received SIRT + Durvalumab + Tremelimumab or TACE + Durvalumab + Tremelimumab.

The other study (NCT04988945), conducted at Queen Mary Hospital (Hong Kong), involves the sequential administration of TACE and SBRT with Durvalumab + Tremelimumab. Table 4 describes current and ongoing studies of combination treatment and immunotherapy for intermediate stage.

## 3. Conclusions and Future Perspective

In summary, we briefly examined the role of the immune system in the development of HCC and the application of checkpoint inhibitors in the field of HCC. We analyzed the completed and ongoing studies testing immunotherapy in the early and intermediate stages of liver cancer and their potential association with local resection and locoregional treatments. However, to date, many questions remain unanswered, such as molecular and immune mechanisms responsible for progression and resistance, the sequences and timing of ICI administration, and which locoregional treatments benefit most from immunotherapeutic interventions. Interdisciplinary efforts of hepatologists, oncologists, biologists, immunologists, and radiologists and carefully conducted trials will make it possible to define the role of ICI in the treatment of early and intermediate stage HCC.

## Figures and Tables

**Table 1:** Ongoing studies of neoadjuvant therapies investigating the combination of ICIs and curative treatments.

Identifier	Agent	Study Type	Outcome Measures	Recruitment Status	Primary Completion Date
NCT04965714	Nivolumab and Pegargiminase Before Resectable HCC	Interventional Phase 2	AEs Rate of pathologic CR	Not yet recruiting	31 March 2022
NCT03299946	Feasibility and Efficacy of Cabozantinib Plus Nivolumab (CaboNivo) Followed by Resection	Interventional Phase 1	AEs Number of patients who complete pre-op treatment and proceed to surgery	Active, not recruiting	9 December 2019
NCT03682276	Safety and Bioactivity of Ipilimumab and Nivolumab Before Resection HCC	Interventional Phase 1, 2	Delay to surgery AEs	Recruiting	1 September 2022
NCT03630640	Neoadjuvant Nivolumab with Electroporation	Interventional Phase 2	RFS Recurrence rate	Recruiting	November 2023
NCT03299946	Feasibility and Efficacy of Cabozantinib Plus Nivolumab (CaboNivo) Followed by Resection	Interventional Phase 1	AEs Number of patients who complete pre-op treatment and proceed to surgery	Active, not recruiting	9 December 2019
NCT04658147	Nivolumab With or Without Relatlimab in Resectable HCC	Phase 1	Number of patients who complete pre-op treatment and proceed to surgery	Recruiting	1 June 2025
NCT04721132	Atezolizumab and Bevacizumab Before Surgery	Interventional Phase 2	pCR rate AEs	Not yet recruiting	30 December 2022
NCT04380545	Nivolumab, Fluorouracil, and Interferon Alpha 2B for Unresectable Fibrolamellar Cancer	Interventional Phase 1–2	AEs	recruiting	20 July 2023
NCT03337841	Pembrolizumab in preventing recurrence before surgery or ablation	Interventional Phase 2	One-year RFS	Unknown	31 October 2019

HCC: Hepatocellular Carcinoma; ICIs: immune checkpoint inhibitors; AEs: adverse events; CR: complete response; RFS: Recurrence-free survival; OS: overall survival; TTR Time to recurrence; RFS: recurrence-free survival; pCR: Pathologic complete response.

**Table 2:** Ongoing studies of adjuvant therapies investigating the combination of ICIs and curative treatments.

Identifier	Agent	Study Type	Outcome Measures	Recruitment Status	Primary Completion Date
NCT03847428	Assess Efficacy and Safety of Durvalumab Alone or plus Bevacizumab After Curative Treatment (EMERALD-2)	Interventional Phase 3	RFS	Recruiting	31 May 2023
NCT03859128	Toripalimab or Placebo After Radical Resection (JUPITER 04)	Interventional Phase 2, 3	RFS	Active, not recruiting	18 April 2023
NCT03383458	Nivolumab After Resection or Ablation	Interventional Phase 3	RFS OS TTR	Active, not recruiting	16 January 2023
NCT03630640	Adjuvant Nivolumab with Electroporation	Interventional Phase 2	RFS Recurrence rate	Recruiting	November 2023
NCT04981665	TACE Sequential Tislelizumab as Adjuvant Therapy After Curative Resection	Interventional Phase 2	2-year RFS Rate	Recruiting	December 2024
NCT04102098	Atezolizumab plus bevacizumab After resected or ablated HCC	Interventional Phase 3	RFS	Active, not recruiting	28 September 2023
NCT03867084	Safety and Efficacy of Pembrolizumab Versus Placebo After Surgical Resection or Local Ablation (MK-3475-937/KEYNOTE-937)	Interventional Phase 3	RFS OS	Recruiting	30 June 2025
NCT04682210	Sintilimab Plus Bevacizumab After Curative Resection	Interventional Phase 3	RFS	Not yet recruiting	December 2023

HCC: Hepatocellular Carcinoma; ICIs: immune checkpoint inhibitors; AEs: adverse events; CR: complete response; RFS: Recurrence-free survival; OS: overall survival; TTR Time to recurrence; RFS: recurrence-free survival; pCR: Pathologic complete response.

**Table 3:** Current and ongoing studies of immunotherapy for intermediate stage.

Identifier	Agent	Study Type	Endpoints	Recruitment Status	Primary Completion Date
NCT04777851	Regorafenib plus Nivolumab Versus TACE in Beyond Up-to-7 (RENOTACE)	Interventional Phase 3	PFS	Recruiting	15 December 2024
NCT04803994	Efficacy and safety of atezolizumab plus bevacizumab plus TACE Versus TACE (ABC-HCC)	Interventional Phase 2	Time to failure of treatment strategy	Recruiting	1 April 2023

HCC: Hepatocellular Carcinoma; ICIs: immune checkpoint inhibitors; SBRT: Stereotactic body radiation therapy SBRT; TATE: Trans-arterial Tirapazamine Embolization; TACE: Transarterial Chemoembolization; OS: Overall Survival; DoR: Duration of response; ORR: Objective response rate; PFS: Progression-free Survival; CRR: Complete Response Rate; RR: Response Rate; TTTP: Time to TACE Progression; AEs: adverse events.

**Table 4:** Current and ongoing studies of combination treatment and immunotherapy for intermediate stage.

Identifier	Agent	Study Type	Endpoints	Recruitment Status	Primary Completion Date
NCT04174781	Neoadjuvant Therapy for Hepatocellular Carcinoma	Interventional Phase 2	PFS	Recruiting	30 November 2020
NCT04268888	Nivolumab plus TACE/TAE (TACE-3)	Interventional Phase 2–3	OS TTTP	Recruiting	June 2025
NCT04340193	Nivolumab and Ipilimumab plus TACE (CheckMate 74W)	Interventional Phase 3	TTTP OS	Active, not recruiting	28 January 2024
NCT04229355	DEB-TACE plus Lenvatinib or Sorafenib or PD-1 Inhibitor for Unresectable HCC	Interventional Phase 3	PFS	Recruiting	30 December 2022
NCT04246177	Lenvatinib and pembrolizumab plus TACE	Interventional Phase 3	PFS OS	Recruiting	25 April 2025
NCT04472767	Cabozantinib plus Ipilimumab/Nivolumab and TACE	Interventional Phase 2	Percentage of Participants with PFS CRR	Recruiting	1 March 2022
NCT04975932	Efficacy and Safety of TACE plus ICIs	Observational	PFS	Recruiting	1 October 2021
NCT04522544	Durvalumab (MEDI4736) and Tremelimumab plus Y-90 SIRT or TACE	Interventional Phase 2	ORR at 6 months	Recruiting	31 March 2024
NCT04988945	TACE and SBRT followed by Durvalumab or Tremelimumab	Interventional Phase 2	Downstaging for hepatectomy	Recruiting	1 December 2022
NCT03638141	Durvalumab and Tremelimumab Following DEB-TACE	Interventional Phase 2	ORR	Recruiting	November 2023
NCT03572582	TACE plus Nivolumab	Interventional Phase 2	ORR	Active, not recruiting	June 2023
NCT03033446	Y90-Radioembolization plus Nivolumab	Interventional Phase 2	RR	Active, not recruiting	December 2021
NCT03778957	TACE plus Durvalumab and Bevacizumab (EMERALD-1)	Interventional Phase 3	PFS	Recruiting	19 September 2022
NCT03397654	Pembrolizumab Following TACE (PETAL)	Interventional Phase 1b	AEs	Recruiting	31 December 2021
NCT05063565	TheraSphere +/− Durvalumab and Tremelimumab (ROWAN)	Interventional Phase 2	ORR Complete response and partial response DoR	Not yet recruiting	June 2025
NCT04712643	Efficacy and Safety of atezolizumab plus bevacizumab plus TACE Versus TACE	Interventional Phase 3	TACE PFS OS	Recruiting	28 February 2025

HCC: Hepatocellular Carcinoma; ICIs: immune checkpoint inhibitors; SBRT: Stereotactic body radiation therapy SBRT; TATE: Trans-arterial Tirapazamine Embolization; TACE: Transarterial Chemoembolization; OS: Overall Survival; DoR: Duration of response; ORR: Objective response rate; PFS: Progression-free Survival; CRR: Complete Response Rate; RR: Response Rate; TTTP: Time to TACE Progression; AEs: adverse events.
